# Skin Closure Technique and Postprocedural Pain after Spinal Cord Stimulator Implantation: A Retrospective Review

**DOI:** 10.1155/2021/9912861

**Published:** 2021-06-04

**Authors:** Markus A. Bendel, Ryan S. D'Souza, Taylor J. North, Thomas P. Pittelkow, Jonathan M. Hagedorn

**Affiliations:** ^1^Department of Anesthesiology and Perioperative Medicine, Division of Pain Medicine, Mayo Clinic, Rochester, MN 55905, USA; ^2^Department of Physical Medicine and Rehabilitation, Mayo Clinic, Rochester, MN 55905, USA

## Abstract

Spinal cord and dorsal root ganglion stimulation are minimally invasive surgical techniques used to treat an array of chronic pain disorders. There is a paucity of data related to defining best practices in these specific patient populations, and historically, providers have relied on consensus committees to opine on the best techniques for patient safety and experience. The most efficacious mechanism of surgical closure—specifically a running suture closure compared to a surgical staple closure—is debated. A retrospective review of 155 patients implanted with either a spinal cord or dorsal root ganglion stimulator between 2017 and 2019 was undertaken to determine if the type of surgical closure was related to degree of postoperative surgical site discomfort. The primary outcome showed no statistically significant difference on postoperative pain scores between the suture (6.0 (IQR 5.0–8.0)) and staple (7.0 (IQR 5.0–8.0)) cohorts at postoperative day (POD) #1 (adjusted *β* 0.17 (95% CI −0.61 to 0.95), *P*=0.670). This finding held for postoperative pain scores at POD #10 as well (staples (1.0 (IQR 0.0–4.0)) and suture (2.0 (IQR 0.0–5.0), adjusted *β* −0.39 (95% CI −1.35 to 0.58), *P*=0.432)). A regression analysis was performed to identify secondary factors impacting postoperative pain scores. Higher preoperative pain score (*β* 0.50 (95% CI 0.09 to 0.92), *P*=0.019) and female gender (*β* 1.09 (95% CI 0.15 to 2.02), *P*=0.023) were predictive of higher incisional pain scores at POD#10. Increasing age was associated with decreased incisional pain scores at POD#10 (*β* −0.06 (95% CI −0.09 to −0.03), *P* < 0.001). These findings are of interest to the pain practitioner and may be valuable in preoperative discussions with prospective patients.

## 1. Introduction

Spinal cord stimulation (SCS) and dorsal root ganglion stimulation (DRG-S) are minimally invasive surgical approaches that are commonly used for certain refractory pain conditions [1–4]. The expanding indications, progressive improvement in hardware, and development of new programming options have resulted in an increasing number of neuromodulation operations [5–7]. The surgical technique for implantation of a percutaneous SCS or DRG-S device generally involves the creation of multiple incisions: one utilized to insert and anchor the stimulating lead (s) and another to insert the implantable pulse generator (IPG). Of course, these incisions require proper surgical closure to prevent wound breakdown, limit infection, and provide optimal postoperative cosmesis. National consensus guidelines have been published to report best practices related to the utilization of SCS and DRG-S therapies. Current specialty guidelines call for skin closure after implantation to be done with either subcuticular suture or staples as the existing evidence does not clearly support either method as superior [8, 9].

Data from other specialties have been published to look for differences in cosmesis, surgical site infection, and patient satisfaction between the two closure methods [10–15]. When these reviews are taken in sum, there is much conflicting data and no clear evidence as to the best technique. However, there may be differences related to a given specific surgical procedure. To our knowledge, there are no data directly comparing skin closure type for SCS and DRG-S surgeries. It is possible that patients who are suffering from chronic pain respond differently to the different closure modalities given the underlying sensitized peripheral and central neuropathic pain pathways. Interest in this simple question is derived from our busy surgical practice, where anecdotally, the clinical support teams at the authors' institution frequently report that stapled closures result in a more painful postoperative recovery for the patient. Based on our clinical and surgical experience, we believe that the skin closure when performed properly does not directly affect patient reported postoperative surgical site pain.

The primary aim of this retrospective study is to provide data from the SCS and DRG-S implant population regarding the impact of closure type, specifically skin staples versus running subcuticular Monocryl, on patient-reported postoperative incisional pain. Second, this study will attempt to isolate patient factors that are predictive of higher postoperative pain scores.

## 2. Materials and Methods

### 2.1. Study Design and Patient Selection

This study was reviewed and approved by the corresponding author's Institutional Review Board as an exempt study. This was a retrospective comparative cross-sectional study that included 155 patients who were seen in the chronic pain clinic at a tertiary referral center (Mayo Clinic, Rochester, MN) between January 1, 2017, and December 31, 2019. Patients >18 years old who underwent percutaneous SCS or DRG-S implantation were identified. Patients were only included if they provided either a postoperative day (POD) #1 incisional pain score or a POD#10 incisional pain score. This strategy was selected to maximize our sample size for analysis. The need for consent was waived by the IRB.

### 2.2. Data Collection

All patients' records were reviewed retrospectively by a single, unbiased individual (TJN). Data that were abstracted included age, sex, body mass index (BMI), overall preprocedural pain score (not for incisional site), POD#1 pain score at incisional site of implant, POD#10 pain score at incisional site of implant, history of fibromyalgia, history of preprocedural opioid use, type of implant (dorsal column SCS or DRG-S), device vendor, and type of suture material used (staples or running subcuticular 4-0 Monocryl suture). The type of closure utilized is dictated by surgeon' preference at the time of surgery. The 11-point numeric rating scale (NRS) was used to rate pain scores, with a score of 0 indicating “no pain” and score of 10 indicating the “worst possible pain.”

### 2.3. Reporting of Demographic and Baseline Clinical Data

Variables were summarized by median and interquartile range (25–75 percentile) for continuous outcomes and frequency (%) for categorical outcomes. Mean and standard deviation were not presented for continuous data as they were not normally distributed. Demographic characteristics and other clinical variables were reported separately for patients that provided a preprocedural NRS score and a POD#1 score or a preprocedural NRS score and a POD#10 score.

### 2.4. Primary Outcome of Interest and Statistical Analysis

The primary outcome of interest was comparison of POD#1 and POD#10 NRS pain scores between patients who received staples versus patients who received running suture for superficial skin closure. The two cohorts (staples and suture cohort) in our overall sample were not matched on a case-by-case basis, and there was concern for results being impacted by various confounding variables. Therefore, linear regression analysis with adjustment for selected confounding variables was chosen as the preferable analysis method. Both unadjusted and adjusted linear regression models were fitted for the primary outcome. Adjusted linear regression controlled for age, sex, BMI, preprocedural pain score, type of implant, history of opioid use, and history of fibromyalgia. These variables were selected a priori for adjustment in the regression model based on author expertise and experience in potential confounding variables that may impact pain-related outcomes. This was also based on prior literature highlighting potential risk factors that may impact postprocedural pain scores across a variety of pain-related procedures [16, 17].

For linear regression models, we reported *β*-coefficients and 95% confidence interval (CI). A *P* < 0.05 was considered the threshold for statistical significance for all comparisons. All analyses, including the primary outcome and secondary outcomes, were performed using SPSS (IBM SPSS Statistics for Windows, Version 21.0, Armonk, NY: IBM Corp.).

### 2.5. Secondary Outcomes of Interest and Statistical Analysis

Secondary outcome analysis included identification of patient risk factors and postoperative pain scores for POD#1 and POD#10. Similar to the primary outcome of interest, this was performed separately for patients that provided a preprocedural NRS score and a POD#1 score and patients that provided a preprocedural NRS score and a POD#10 score. Unadjusted linear regression models were performed to identify associations between selected risk factors (age, sex, BMI, preprocedural pain score, history of fibromyalgia, and history of preprocedural opioid use) and postoperative pain scores.

An additional secondary outcome included change in postoperative incisional pain scores based on suture technique over time (from POD#1 to POD#10). Since this outcome necessitates inclusion of patients who reported pain scores at all time points, only patients who reported preprocedural pain score, POD#1 incisional pain score, and POD#10 incisional pain score were included in this portion of the secondary outcome analysis. Pearson's correlation coefficient (*r*) was calculated to determine the correlation between incisional pain and time (POD#1 to POD#10). A subgroup analysis of this trend line was also performed noting changes in postoperative incisional pain scores over time based on suture technique as well as sex. Therefore, this subgroup analysis will present change in incisional pain score for females in the staples cohort, females in the suture cohort, males in the staples cohort, and males in the suture cohort separately over time (POD#1 to POD#10).

## 3. Results

### 3.1. Demographic and Baseline Clinical Data

Retrospective review on a total of 155 unique patients was performed. Of these patients, 152 reported POD#1 NRS incisional pain scores and 124 reported POD#10 NRS incisional pain scores. The median age was 65.0 (IQR 52.0–75.0) for the overall cohort that reported POD#1 NRS incisional pain scores and 62.0 (IQR 50.5–74.0) for the overall cohort that reported POD#10 NRS incisional pain scores. Females comprised 50.6% of the overall cohort among those that reported POD#1 NRS incisional pain scores and 52.4% among those that reported POD#10 NRS incisional pain scores.

Demographic characteristics and other clinical variables based on superficial closure technique are presented in Table 1. Comparisons of baseline demographic and clinical variables were not statistically different between cohorts. The continuous outcome data did not follow a normal distribution and therefore are presented using median values with interquartile range. However, for additional information, to inform the reader, we also presented mean and standard deviation values for each continuous variable in Supplementary Table 1.

### 3.2. Primary Outcome

Adjusted and unadjusted linear regression models revealed comparable POD#1 NRS incisional pain scores between patients who received staples for superficial skin closure (7.0 (IQR 5.0–8.0)) versus those who received sutures (6.0 (IQR 5.0–8.0), adjusted *β* 0.17 (95% CI −0.61 to 0.95), *P*=0.670). Similarly, adjusted and unadjusted linear regression models also revealed comparable POD#10 NRS incisional pain scores between the staples cohort (1.0 (IQR 0.0–4.0)) and the suture cohort (2.0 (IQR 0.0–5.0), adjusted *β* −0.39 (95% CI −1.35 to 0.58), *P*=0.432) (Table 2).

### 3.3. Secondary Outcomes

Unadjusted regression analysis was performed for the initial portion of the secondary outcomes to identify risk factors of postoperative incisional pain scores. This analysis identified that severity of preprocedural pain score was associated with greater POD#1 incisional pain scores in the overall cohort (*β* 0.41 (95% CI 0.08 to 0.73), *P*=0.014) as well as the suture cohort (*β* 0.78 (95% CI 0.26 to 1.31), *P*=0.004). Similarly, severity of preprocedural pain score was associated with greater POD#10 incisional pain scores in the overall cohort (*β* 0.50 (95% CI 0.09 to 0.92), *P*=0.019) and the staples cohort (*β* 0.58 (95% CI 0.06 to 1.10), *P*=0.030) (Table 3). As age increased, there was an association with decreased POD#10 incisional pain scores in the overall cohort (*β* −0.06 (95% CI −0.09 to −0.03), *P*=0.001) and the staples cohort (*β* −0.07 (95% CI −0.12 to −0.03), *P*=0.001). Finally, female sex was associated with greater POD#1 incisional pain scores in the overall cohort (*β* 1.19 (95% CI 0.47 to 1.91), *P*=0.001) and the staples cohort (*β* 1.43 (95% CI 0.50 to 2.36), *P*=0.003). Similarly, female sex was associated with greater POD#10 incisional pain scores in the overall cohort (*β* 1.09 (95% CI 0.15 to 2.02), *P*=0.023).

**Figure 1:**
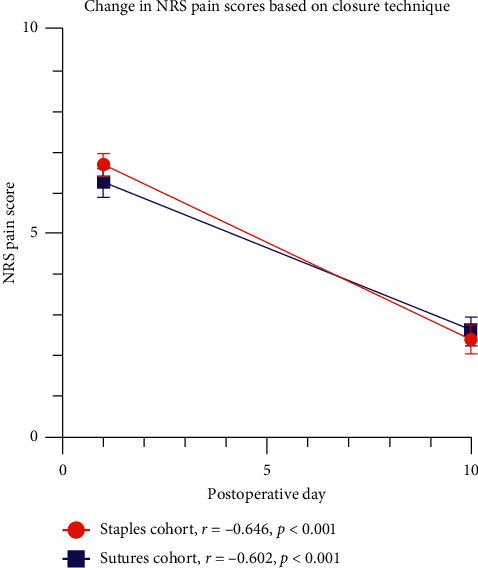
Change in procedural NRS by postoperative day. Change in mean NRS pain score is depicted from POD#1 to POD#10 based on closure technique. The total overall sample that reported all three scores (preprocedural pain score, POD#1 score, and POD#10 score) was 116 patients and were included in this trend line analysis to visualize chance in pain scores over time. Trend line analysis was performed using Pearson's correlation coefficient *r*. On POD#1, the mean ± standard error was 6.7 ± 0.3 (*n* = 68) for the staples cohort and 6.3 ± 0.3 for the sutures cohort (*n* = 48). On POD#10, the mean ± standard error was 2.4 ± 0.3 (*n* = 68) for the staples cohort and 2.6 ± 0.4 for the suture cohort (*n* = 48).

The other secondary outcome of change in postoperative incisional pain scores over time based on suture technique was analyzed using Pearson's correlation coefficient (*r*). When analyzing the correlation of NRS incisional pain scores over time in patients who reported preprocedural scores, POD#1 scores, and POD#10 scores, there was an inverse correlation between NRS incisional pain scores and POD for both the staples cohort (Pearson's *r* = −0.646, *P* < 0.001) and the suture cohort (Pearson's *r* = −0.602, *P* < 0.001) (Figure 1). This was further stratified with a subgroup analysis based on sex and closure technique (Supplementary Figure 1) and similarly displays significant inverse Pearson's correlation coefficients between postoperative incisional pain scores and time.

## 4. Discussion

The primary aim of this retrospective study was to differentiate whether staples or suture-based skin closure results in different postoperative pain scores for patients undergoing implantation of a neuromodulation device. The results from this retrospective review show that there is not a statistically significant difference at either the POD#1 or POD#10 time points between the two cohorts: skin staples versus running Monocryl skin closure. Secondary outcomes were revealing several important findings. First, patients with higher preoperative chronic pain scores at baseline reported higher postoperative procedural pain. In addition, regardless of skin closure type, there was a dramatic improvement in postoperative surgical pain at the ten-day postoperative follow-up time period. Next, female sex was associated with higher reported postoperative pain scores. Finally, as age increased, postoperative pain scores decreased.

Pain is a unique experience that is individualized and can be particularly complex for patients living with chronic pain. Our findings would suggest that when skin closure is performed appropriately, there was not a statistical difference between skin closure type and postoperative incisional pain. This is consistent with other surgical specialty literature. A recent systematic review suggested that the use of sutures slightly reduced the patient's reported postoperative procedural pain; however, significant challenges with heterogeneity were reported and admittedly based on imprecise high confidence interval data [11]. One additional fact to consider is that patient preference may also play a role in perception of pain relief postoperatively. Reported preference of suture versus staples for skin closure is regularly debated, and more often than not, patients prefer the use of suture for the simple reason of avoiding the need for staple removal [18]. In the end, as is outlined in the specialty best practice guidelines for neuromodulation, it appears reasonable to choose either staples or running suture based on surgeon preference and proficiency [8].

Interestingly, some associations between patient characteristics and postoperative incisional pain scores were uncovered. It is important to note that in this dataset, the patients are specifically asked to comment on their procedural/incisional pain as a distinct entity from their chronic pain. This can be a challenging endeavor. The overall cohort showed that patients with a higher preoperative (chronic) pain score had an association of higher postoperative incisional pain scores on POD#1 and POD#10. This is not clinically surprising given the concepts of central sensitization and neurophysiologic pathology of chronic pain, but it is important to recognize this association. It has been well established that chronic pain is a known risk factor for the development of higher acute postoperative pain scores [19]. An important part of the perioperative experience is education of the patient with chronic pain to expect elevated postoperative procedural pain that will improve rapidly within ten days postoperatively. This important rapid decline of postoperative procedural pain was demonstrated in the dataset, which showed quick improvement in incisional pain scores between POD#1 and POD#10 (7.0–1.0 for staples and 6.0–2.0 for sutures). This information can be used to help guide patients' expectations of their surgical experience and wound healing with either suture or staple closure.

When the data were analyzed for possible contribution of gender, it was discovered that female patients reported higher postoperative pain scores. While the exact explanation is indeterminate for this observation, it is an interesting finding. An additional association was that of advancing age and decreased reported pain scores. These are important patterns to understand as the nature of spinal cord stimulation is completely elective. Patient selection is critical for long-term success of the therapy. Being able to properly educate patients on individual risk factors for immediate postoperative pain may be reassuring for patients and providers.

As with all studies, this retrospective review is not without limitations. The primary limitation is attributed to the retrospective nature of the study-causal relationships that cannot be established. Second, the dataset had a small number of patients that did not complete the standard follow-up pathway. Therefore, POD#1 or POD#10 NRS scores were not documented for these patients and were not included in the study sample. Finally, results of a single center experience are not ubiquitous, and there likely are regional differences that factor into a patient's perception of postoperative pain. Ideally, a multicenter randomized controlled trial could be executed to definitively address this question in the SCS and DRG-S populations.

## 5. Conclusion

This retrospective study indicates that there may not be a significant relationship between choice of surgical skin closure and relationship to postoperative incisional pain following implantation of SCS or DRG-S devices. However, several important associations were discovered: a positive association between preoperative pain severity and postoperative incisional pain severity, an association between female sex and higher reported incisional pain scores, and an inverse correlation between age and reported postoperative pain scores. This information can be useful to inform patients and implanting physicians alike on the expected postoperative course and help to predict which patient may have a more painful postoperative course.

## Figures and Tables

**Table 1:** Demographic and other baseline variables based on superficial closure technique.

	Overall cohort (*n* = 152)	Staples (*n* = 88)	Suture (*n* = 64)	*P* value
*Patients reporting preprocedural and POD#1 NRS score*				
Age at implant^a^	65.0 (52.0–75.0)	62.5 (52.2–73.8)	66.0 (52.0–75.0)	0.573
Sex^b^				
Female	77 (50.6)	48 (54.5)	29 (45.3)	0.3244
Male	75 (49.3)	40 (45.5)	35 (54.7)	

BMI^a^	31.2 (27.7–34.6)	31.5 (28.4–35.3)	30.8 (26.0–34.5)	0.196
Preprocedural pain score^a^	8.0 (7.0–9.0)	8.0 (8.0–10.0)	8.0 (8.0–9.0)	0.975
History of fibromyalgia^b^	7 (4.8)	6 (7.2)	1 (1.6)	0.141
History of opioid use^b^	42 (27.6)	24 (27.3)	18 (28.1)	1.000

Implant type^b^				
Dorsal column spinal cord stimulator	133 (87.5)	77 (87.5)	56 (87.5)	1.000
Dorsal root ganglion stimulator	19 (12.5)	11 (12.5)	8 (12.5)	

Vendor company^b^				
Abbott	20 (13.2)	11 (12.5)	9 (14.1)	0.811
Medtronic	62 (40.8)	36 (40.9)	26 (40.6)	1.000
Nevro	70 (46.0)	41 (46.6)	29 (45.3)	1.000

	Overall cohort (*n* = 124)	Staples (*n* = 73)	Suture (*n* = 51)	*P* value
*Patients reporting preprocedural and POD#10 NRS score*				

Age at implant^a^	62.0 (50.5–74.0)	61.0 (48.0–73.0)	64.5 (50.8–75.3)	0.517
Sex^b^				
Female	65 (52.4)	40 (54.8)	25 (49.0)	0.586
Male	59 (47.6)	33 (45.2)	26 (51.0)	

BMI^a^	31.1 (27.7–34.6)	31.2 (28.1–34.4)	30.6 (26.3–34.7)	0.489
Preprocedural pain score^a^	8.0 (8.0–9.2)	8.0 (8.0–10.0)	8.0 (8.0–9.0)	0.808
History of fibromyalgia^b^	7 (5.7)	5 (6.9)	2 (3.9)	0.698
History of opioid use^b^	38 (30.6)	23 (31.5)	15 (29.4)	0.845
Implant type^b^				
Dorsal column spinal cord stimulator	108 (87.1)	64 (87.7)	44 (86.3)	1.000
Dorsal root ganglion stimulator	16 (12.9)	9 (12.3)	7 (13.7)	

Vendor company^b^				
Abbott	17 (13.7)	9 (12.3)	8 (15.7)	0.606
Medtronic	48 (38.7)	29 (39.7)	19 (37.2)	0.852
Nevro	59 (47.6)	35 (47.9)	24 (47.0)	1.000

^a^Median value (25–75% interquartile range); ^b^number (%); different sample sizes are present on postoperative day #1 and postoperative day #10 based on capture of patient report of pain scores. ^*∗*^*P* value <0.05; the Mann–Whitney *U* test was performed to compare continuous outcomes, and Fisher's exact test was performed to compare categorical outcomes.

**Table 2:** Comparison of postoperative incisional pain scores based on superficial closure technique.

	Overall cohort median score (*n* = 152)	Staples cohort median score (*n* = 88)	Suture cohort median score (*n* = 64)	Unadjusted *β*-coefficient (95% CI)	Unadjusted *P* value	Adjusted *β*-coefficient (95% CI)	Adjusted *P* value
POD#1 NRS score^a^	7.0 (5.0–8.0)	7.0 (5.0–8.0)	6.0 (5.0–8.0)	0.44 (−0.32 to 1.19)	0.252	0.17 (−0.61 to 0.95)	0.670

	Overall cohort median score (*n* = 124)	Staples cohort median score (*n* = 73)	Suture cohort median score (*n* = 51)	Unadjusted *β*-coefficient (95% CI)	Unadjusted *P* value	Adjusted *β*-coefficient (95% CI)	Adjusted *P* value

POD#10 NRS score^a^	2.0 (0.0–4.0)	1.0 (0.0–4.0)	2.0 (0.0–5.0)	−0.28 (−1.24 to 0.69)	0.572	−0.39 (−1.35 to 0.58)	0.432

^a^Median value (25–75% interquartile range). Unadjusted and adjusted linear regression was performed to compare pain scores based on superficial closure technique. The *β*-coefficients were performed with the reference cohort being the suture cohort. Variables that were controlled in the adjusted linear model included age, sex, body mass index, preprocedural pain score, type of implant, history of opioid use, and history of fibromyalgia. The *P* value that is provided is based on the adjusted regression model. ^*∗*^*P* value <0.05. POD, postoperative day.

**Table 3:** Association between risk factors and postoperative pain scores.

	Overall cohort *β*-coefficient (95% CI)	*P* value	Staples cohort *β*-coefficient (95% CI)	*P* value	Suture cohort *β*-coefficient (95% CI)	*P* value
Postoperative day #1						
Age at implant	−0.02 (−0.04 to 0.01)	0.180	−0.02 (−0.06 to 0.02)	0.267	−0.01 (−0.05 to 0.02)	0.471
Sex	1.19 (0.47 to 1.91)	0.001^*∗*^	1.43 (0.50 to 2.36)	0.003^*∗*^	1.25 (−0.15 to 2.65)	0.079
BMI	−0.02 (−0.09 to 0.05)	0.520	0.01 (−0.08 to 0.10)	0.792	−0.08 (−0.18 to 0.03)	0.165
Preprocedural pain score	0.41 (0.08 to 0.73)	0.014^*∗*^	0.18 (−0.23 to 0.60)	0.377	0.78 (0.26 to 1.31)	0.004^*∗*^
History of fibromyalgia	1.65 (−0.12 to 3.42)	0.068	1.50 (−0.44 to 3.43)	0.129	1.95 (−2.80 to 6.70)	0.415
History of opioid use	−0.41 (−1.24–0.43)	0.338	−0.85 (−1.93 to 0.22)	0.118	0.21 (−1.13 to 1.55)	0.754

	Overall cohort *β*-coefficient (95% CI)	*P* value	Staples cohort *β*-coefficient (95% CI)	*P* value	Suture cohort *β*-coefficient (95% CI)	*P* value
Postoperative day #10						

Age at implant	−0.06 (−0.09 to −0.03)	<0.001^*∗*^	−0.07 (−0.12 to −0.03)	0.001^*∗*^	−0.04 (−0.09 to 0.01)	0.092
Sex	1.09 (0.15 to 2.02)	0.023^*∗*^	0.95 (−0.34 to 2.25)	0.148	1.32 (−0.058 to 2.70)	0.060
BMI	−0.03 (−0.11 to 0.06)	0.527	−0.01 (−0.12 to 0.11)	0.930	−0.06 (−0.20 to 0.08)	0.381
Preprocedural pain score	0.50 (0.09 to 0.92)	0.019^*∗*^	0.58 (0.06 to 1.10)	0.030^*∗*^	0.31 (−0.44 to 1.06)	0.414
History of fibromyalgia	0.98 (−1.08 to 3.04)	0.348	0.43 (−2.17 to 3.02)	0.744	2.45 (−1.16 to 6.06)	0.179
History of opioid use	−0.58 (−1.61 to 0.44)	0.263	−0.16 (−1.57 to 1.25)	0.822	−1.20 (−2.73 to 0.33)	0.121

Linear regression models were performed to identify associations between selected risk factors and postoperative pain. This is presented for the overall cohort as well as stratified based on staples and suture. ^*∗*^*P* value <0.05.

## Data Availability

The data used to support the findings of this study have not been made available due to patient confidentiality.

## Supplementary Materials

Supplementary Materials Supplementary Table 1. Mean demographic and outcome variables. Supplementary Figure 1. Change in procedural NRS by postoperative day-sex subgroup analysis.
