# Supplementary material for: Skin Closure Technique and Postprocedural Pain after Spinal Cord Stimulator Implantation: A Retrospective Review
Source: Pain Res Manag. 2021 Jun 4;2021:9912861. doi: 10.1155/2021/9912861 (PMC8195651; doi:10.1155/2021/9912861)
Supplement: Supplementary Materials — Supplementary Table 1. Mean demographic and outcome variables. Supplementary Figure 1. Change in procedural NRS by postoperative day-sex subgroup analysis. [file 9912861.f1.zip › 9912861.f1/FinalSuppFigure1.docx]

**Supplemental Figure 1.** Change in procedural NRS by post-operative day - sex subgroup analysis


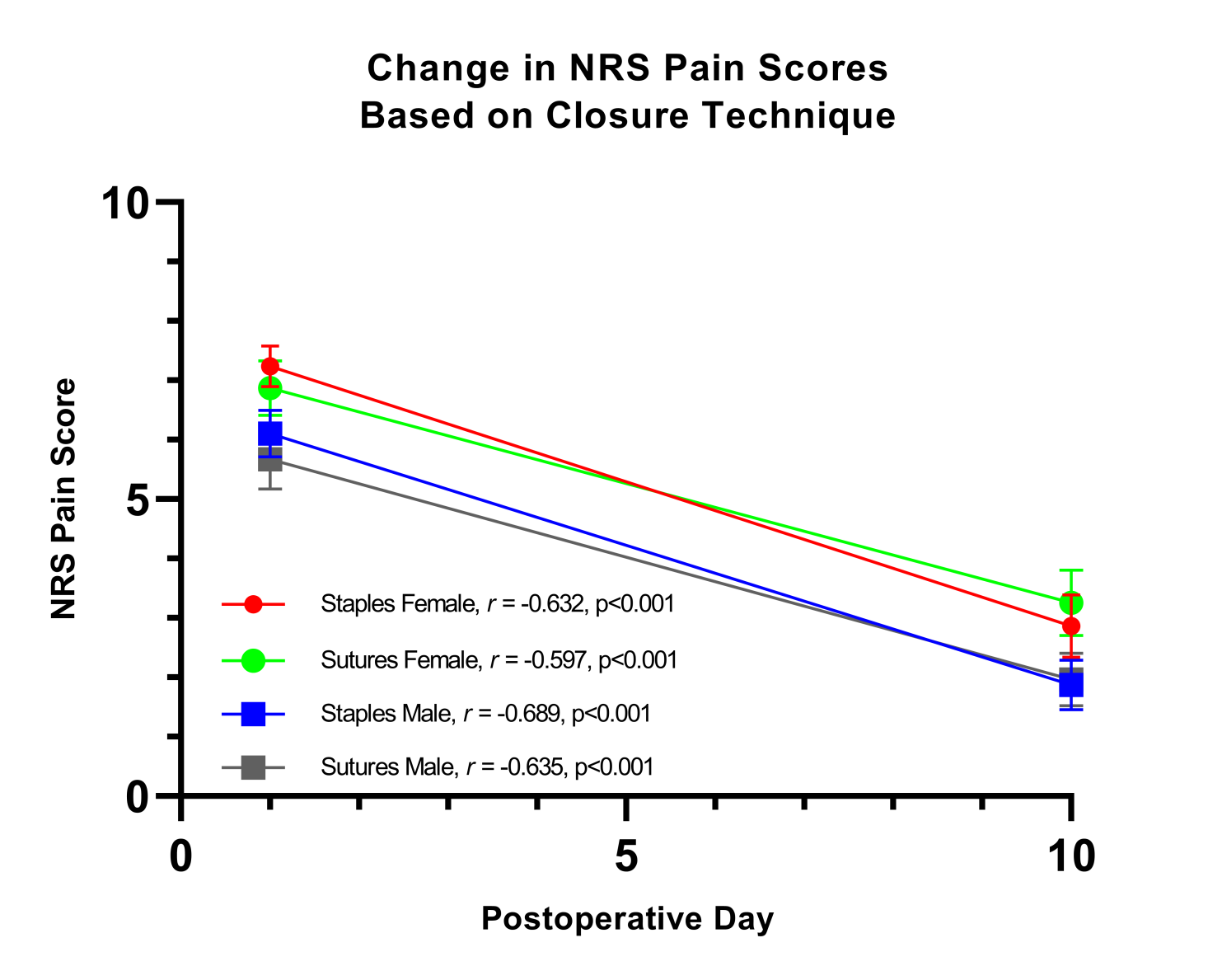


Change in mean NRS pain score is depicted from POD#1 to POD#10 based on closure technique and sex. Trend line analysis was performed using Pearson’s correlation coefficient (*r*). On POD#1 for females, the mean ± standard error was 7.2 ± 0.3 (n=37) for the staples cohort, and 6.9 ± 0.5 (n=24) for the sutures cohort. On POD#10 for females, the mean ± standard error was 2.9± 0.5 (n=37) for the staples cohort, and 3.2 ± 0.6 (n=24) for the suture cohort. On POD#1 for males, the mean ± standard error was 6.1 ± 0.4 (n=31) for the staples cohort, and 5.7 ± 0.5 (n=24) for the sutures cohort. On POD#10 for males, the mean ± standard error was 1.9 ± 0.4 (n=31) for the staples cohort, and 2.0 ± 0.4 (n=24) for the suture cohort.
