# Supplementary material for: Skin Closure Technique and Postprocedural Pain after Spinal Cord Stimulator Implantation: A Retrospective Review
Source: Pain Res Manag. 2021 Jun 4;2021:9912861. doi: 10.1155/2021/9912861 (PMC8195651; doi:10.1155/2021/9912861)
Supplement: Supplementary Materials — Supplementary Table 1. Mean demographic and outcome variables. Supplementary Figure 1. Change in procedural NRS by postoperative day-sex subgroup analysis. [file 9912861.f1.zip › 9912861.f1/FinalSuppTable1.docx]

| **Supplemental Table 1. Mean Demographic and Outcome Variables** | | |
| --- | --- | --- |
|  | **Staples (n=88)** | **Suture (n=64)** |
| **Patients Reporting Pre-procedural and POD #1 NRS Score** | | |
| Age at Implant | 61.9±14.5 | 63.3±15.2 |
| BMI | 31.9±5.6 | 30.4±5.4 |
| Pre-procedural Pain Score | 8.5±1.1 | 8.5±1.1 |
| Post-procedural Pain Score POD#1 | 6.4±2.3 | 6.1±2.4 |
|  | **Staples (n=73)** | **Suture (n=51)** |
| **Patients Reporting Pre-procedural and POD #10 NRS Score** | | |
| Age at Implant | 61.2±14.7 | 63.4±15.0 |
| BMI | 31.5±5.7 | 30.6±5.3 |
| Pre-procedural Pain Score | 8.5±1.2 | 8.6±1.0 |
| Post-procedural Pain Score POD#10 | 2.4±2.8 | 2.6±2.5 |

Mean and standard deviations are provided for continuous variables. Given that these were not normally distributed data, median values and interquartile range are presented in Tables 1 and 2, which should be considered the primary analysis.
