# Supplementary material for: Skin Closure Technique and Postprocedural Pain after Spinal Cord Stimulator Implantation: A Retrospective Review
Source: Pain Res Manag. 2021 Jun 4;2021:9912861. doi: 10.1155/2021/9912861 (PMC8195651; doi:10.1155/2021/9912861)
Supplement: Supplementary Materials — Supplementary Table 1. Mean demographic and outcome variables. Supplementary Figure 1. Change in procedural NRS by postoperative day-sex subgroup analysis. [file 9912861.f1.zip › 9912861.f1/Supplementary Descriptions.docx]

**Supplementary Descriptions**

Supplemental Table 1. Mean Demographic and Outcome Variables

Supplemental Figure 1. Change in procedural NRS by post-operative day - sex subgroup analysis
